# Chromatin accessibility profiling in *Neurospora crassa* reveals molecular features associated with accessible and inaccessible chromatin

**DOI:** 10.1186/s12864-021-07774-0

**Published:** 2021-06-19

**Authors:** Aileen R. Ferraro, Abigail J. Ameri, Zefu Lu, Masayuki Kamei, Robert J. Schmitz, Zachary A. Lewis

**Affiliations:** 1 Department of Microbiology, University of Georgia, Athens, GA 30602 USA; 2 Department of Genetics, University of Georgia, Athens, GA 30602 USA

**Keywords:** Chromatin accessibility, ATAC-seq, Fungi, H3K36me3

## Abstract

**Background:**

Regulation of chromatin accessibility and transcription are tightly coordinated processes. Studies in yeast and higher eukaryotes have described accessible chromatin regions, but little work has been done in filamentous fungi.

**Results:**

Here we present a genome-scale characterization of accessible chromatin regions in *Neurospora crassa*, which revealed characteristic molecular features of accessible and inaccessible chromatin. We present experimental evidence of inaccessibility within heterochromatin regions in *Neurospora,* and we examine features of both accessible and inaccessible chromatin, including the presence of histone modifications, types of transcription, transcription factor binding, and relative nucleosome turnover rates. Chromatin accessibility is not strictly correlated with expression level. Accessible chromatin regions in the model filamentous fungus *Neurospora* are characterized the presence of H3K27 acetylation and commonly associated with pervasive non-coding transcription. Conversely, methylation of H3 lysine-36 catalyzed by ASH1 is correlated with inaccessible chromatin within promoter regions. **Conclusions:** In *N. crassa,* H3K27 acetylation is the most predictive histone modification for open chromatin. Conversely, our data show that H3K36 methylation is a key marker of inaccessible chromatin in gene-rich regions of the genome. Our data are consistent with an expanded role for H3K36 methylation in intergenic regions of filamentous fungi compared to the model yeasts, *S. cerevisiae* and *S. pombe,* which lack homologs of the ASH1 methyltransferase.

**Supplementary Information:**

The online version contains supplementary material available at 10.1186/s12864-021-07774-0.

## Background

Functionally distinct chromatin environments are defined by characteristic molecular features including histone modifications, occupancy of DNA- and chromatin-binding proteins, and varying degrees of chromatin accessibility (for a review, see [1]). Euchromatin is traditionally defined as gene-rich, transcriptionally active and enriched for specific chromatin modifications including histone H3 acetylation (H3Kac), histone H3 lysine 4 methylation (H3K4me1/2/3), and histone H3 lysine 36 trimethylation (H3K36me3) [2–6]. Conversely, heterochromatin is transcriptionally repressed and marked by histone H3 lysine 9 methylation (H3K9me3) [6–10] or histone H3 lysine 27 methylation (H3K27me2/3) [6, 11–16]⁠. Chromatin environments can also be defined by the presence of histone variants such as histone H2A.Z, which is found surrounding transcriptional start sites and within vertebrate and plant enhancers [17–23].

Euchromatic and heterochromatic environments display marked differences in the level of chromatin accessibility to DNA-binding proteins or other regulatory factors. The Assay for Transposase-Accessible Chromatin sequencing (ATAC-seq) method has proven to be useful tool for characterizing chromatin accessibility in eukaryotes [24]. ATAC-seq has been applied in yeasts, animals, and plants to identify promoter and enhancer environments [24–27]⁠; however, work in non-yeast fungi is limited. A variety of factors appear to contribute to accessible chromatin. For example, certain DNA sequences are thought to exclude nucleosomes and thus contribute to nucleosome depletion near the start of transcription [28]. In addition, binding of pioneer transcription factors within promoter regions can drive the formation of an accessible chromatin environment and promote transcriptional activity [29–32]. In *Neurospora*, previous MNase experiments have shown that binding of the light-response master regulator white collar complex (WCC) subunit white collar 2 (*wc-2*) can change nucleosome organization at promoters of WCC-inducible genes [33]⁠⁠. Additionally, application of ATAC-seq in the pathogenic basidiomycete *Cryptococcus neoformans* has demonstrated that transcription factor ZNF2 and the Switch/Sucrose non-fermentable (SWI/SNF) complex create chromatin accessibility at filamentation genes during filamentation [27]⁠. Another factor that may contribute to accessible chromatin is pervasive non-coding transcription [34–36]. Indeed, a recent study investigated histone modifications and small RNAs at *cis*-regulatory elements in cnidarians, fungi, and human cells, providing evidence that small RNAs or RNA polymerase activity are evolutionary conserved features of accessible chromatin [37]⁠⁠⁠. While this study characterized small RNA production in the filamentous fungus *Neurospora,* raising the possibility that transcription may contribute to accessible chromatin, the authors did not identify accessible chromatin regions in this fungus.

Other chromatin features are correlated with inaccessible chromatin. Constitutive heterochromatin domains marked by H3K9me3 and H3K27me3 are reported to be largely inaccessible in higher eukaryotes [38], but chromatin inaccessibility within these regions has not yet been directly measured in filamentous fungi. In *Saccharomyces cerevisiae,* methylation of H3K36 is mediated by a single histone methyltransferase, SET2, which associates with elongating RNA polymerase to methylate H3K36 in coding sequences [39–42]. Although H3K36me3 occurs in active genes, this modification promotes inaccessible chromatin via recruitment of chromatin remodelers and histone deacetylases that act to repress spurious intergenic transcription from cryptic promoters [43–47]⁠. In certain filamentous fungi and in higher eukaryotes, H3K36 methylation is catalyzed by multiple lysine methyltransferase enzymes. *Neurospora* and the closely related *Fusarium fujikuroi* encode two H3K36 methyltransferases that have been genetically characterized (SET-2 and ASH1) [48–50]. Like yeast SET2, *Neurospora* SET-2 trimethylates H3K36 along gene bodies and is presumably targeted by elongating RNA polymerase [48]⁠. *Neurospora* ASH1, instead, deposits mono- and di-methylation on H3K36 residues across promoters and gene bodies of silent genes in a transcriptionally independent manner [50]⁠⁠. Some regions of ASH1 catalyzed H3K36me2 co-localize with H3K27me2/3 and contribute to Polycomb Repressive Complex 2 (PRC2)-mediated repression [50].

Here, we report the development of an ATAC-seq protocol for filamentous fungi. Using ATAC-seq, we carried out a comprehensive analysis of chromatin features associated with accessible chromatin. We report a diversity of promotor structures in *Neurospora* and we show that histone acetylation and small RNA production are highly correlated with accessible chromatin, whereas repressive modifications including H3K9me3, H3K27me2/3 and ASH1-catalyzed H3K36 methylation are correlated with inaccessible chromatin in this fungus.

## Results

### Characterization of accessible chromatin regions in *Neurospora*

To characterize accessible regions in *Neurospora*, we performed ATAC-seq on wild type mycelial cultures. Because this assay is sensitive to contamination by mitochondria, we first performed ATAC-seq in nuclei that were sorted by flow cytometry ⁠and nuclei that were not sorted. In both cases, sequence reads mapped to the *Neurospora* genome assembly at > 98.4% (Table S1). Fewer reads mapped to the mitochondrial genome in sorted nuclei (~ 15%) when compared to unsorted nuclei (~ 27%) (Fig. S1A; Table S1). We next examined the distribution of ATAC-seq reads from sorted versus unsorted nuclei. We first identified accessible chromatin regions (ACRs) using Genrich from wild type sorted and unsorted nuclei (using standard parameters: minimum area under the curve of 200 bp, significance threshold > 1, and *p*-value < 0.01). We identified 7132 ACRs unsorted cells and 5181 ACRs sorted cells from the wild-type strain. Of the ACRs we identified in sorted cells, 92% overlapped with ACRs called in unsorted cells by bedtools intersect (*n* = 4772; Table S2). We then plotted ATAC-seq enrichment in sorted and unsorted cells centered on the ACRs from unsorted nuclei. Comparison of these two experiments revealed that global patterns of chromatin accessibility in *Neurospora* is unchanged in sorted and unsorted nuclei (Fig. S1B). This is confirmed with an example genome browser image comparing ATAC-seq reads of sorted and unsorted nuclei (Fig. S1C). Based on these data, we concluded that sorting nuclei removed some mitochondrial contamination, but also reduced the sensitivity of the ATAC-seq assay. Thus, it is more efficient and cost-effective to carry out ATAC-seq analysis using unsorted nuclei followed by downstream removal of mitochondrial reads in silico. All remaining experiments were performed with unsorted nuclei.

**Fig. 1:**
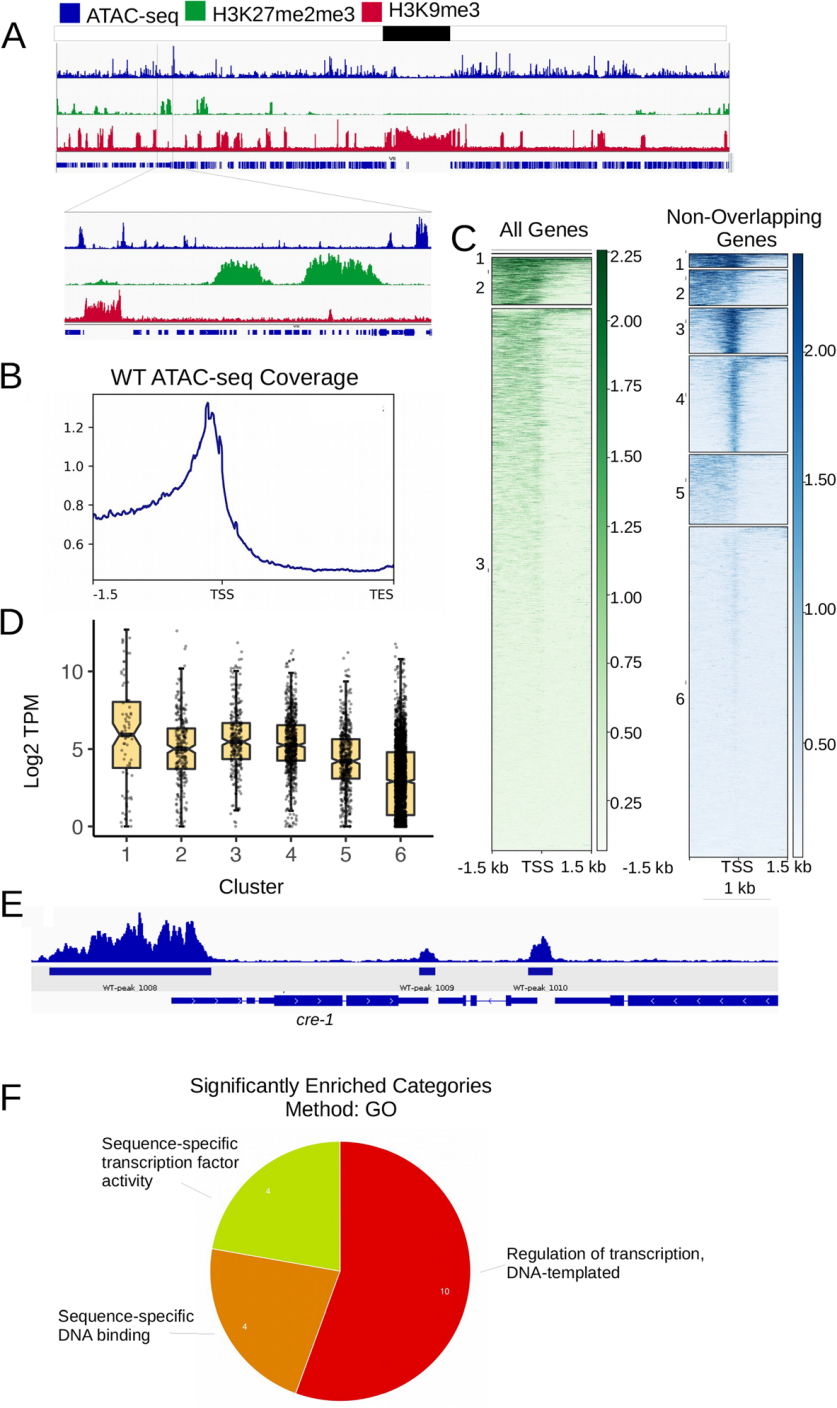
Accessible chromatin is present in gene promoters and absent from heterochromatin. (A) A genome browser image showing ATAC-seq (blue), H3K27me3 (green) and H3K9me3 (red) localization across Linkage Group VII for the wild-type strain. (B) The metaplot shows enrichment of ATAC-seq experiments averaged across all *Neurospora* genes. (C) The heatmaps show the enrichment of ATAC reads in a 3000 bp window centered on the transcriptional start site for all genes (left) or genes with unambiguous promoters (right). Genes were grouped using k-means clustering (left; k = 3; right; k = 6) to illustrate the distinct patterns of chromatin accessibility found in *Neurospora* promoters. (D) Box plots depict the log2-transformed expression levels for genes with unambiguous promoters in the 6 clusters of from panel C (log_2_ [TPM + 1]). The expression levels of individual genes in each cluster are plotted as points. (E) A genome browser shot of the *cre-1* (carbon catabolite repression) gene illustrates an example gene with a large regulatory domain of accessible chromatin (2520 bp). (F) GO analysis shows enrichment for DNA binding and transcription factor activity of genes with large intergenic hyperaccessible domains

We next wanted to determine what chromatin environments contain high enrichment of accessible chromatin. To do this, we first visually inspected ATAC-seq enrichment across the *Neurospora* genome using the Integrated Genomics Viewer (IGV) [51]. We found that chromatin accessibility is highest in intergenic regions and limited or absent in gene bodies (Fig. 1A). Additionally, facultative and constitutive heterochromatin marked by H3K27me2/3 or H3K9me3, respectively, have low levels of chromatin accessibility (Fig. 1A).

To quantify the level of accessibility in heterochromatin and euchromatin, we used bedtools intersect to determine the number of ACRs that overlap with H3K27me2/3 or H3K9me3 (Table S3). H3K27me2/3 is enriched in 309 domains covering 6% of the genome [22]⁠. We identified 68/7132 ACRs that overlap with called H3K27me2/3 enriched regions. The average density of ACRs in these domains is 0.27 ACRs per 10 kb. We also wanted to quantify the number and density of ACRs in constitutive heterochromatin marked by H3K9me3. In total, 454 domains are enriched for H3K9me3 covering approximately 18% of the genome [52]. 198 ACRs overlapped with H3K9me3 with an average peak density of 0.32 ACRs per 10 kb. The remaining 6866 ACRs are found in regions that lack H3K9me3 or H3K27me2/3, with an average density in euchromatin of 2.25 ACRs per 10 kb.

In general, accessible chromatin regions appeared to be restricted to promoter regions of *Neurospora* genes (Fig. 1A). To determine if accessibility was a global feature of promoters, we averaged ATAC-seq data across all promoters and plotted the distribution (Fig. 1B). This confirmed that ACRs are present in the 5′ regions of genes. We next wanted to ask if *Neurospora* promoters exhibit distinct patterns of chromatin accessibility. We did this by performing k-means clustering and plotting a heatmap of ATAC-seq read enrichment across 3000 bp (+/− 1500 bp) of DNA centered on the transcription start site (TSS) of each *Neurospora* gene (Fig. 1C). It was difficult to resolve organization of accessible regions at the single-gene level because the *Neurospora* genome is highly compact; thus, many intergenic regions contain a second promoter within the 3000 base pair window plotted for each gene (e.g. divergently transcribed genes share the same intergenic space; Fig. 1C, Left). To better ascertain the diversity of accessible chromatin structures in *Neurospora* gene regulatory regions, we examined the distribution of ATAC-seq reads only for promoters that could be unambiguously assigned to a single gene (i.e. gene promoters without another annotated transcriptional start site within 1500 base pairs in either direction, which is the average intergenic space in *Neurospora* (Table S4; see Materials and Methods) [53]⁠⁠. We performed k-means clustering and plotted ATAC-seq read enrichment across the the TSSs of this gene set to group genes with similar patterns of chromatin accessibility within promoters and 5′ intergenic regions (Fig. 1C, Right; k = 6). This analysis reveals that the size and pattern of accessible chromatin varies for genes throughout the *Neurospora* genome. For example, Cluster 1 and 2 show broad, highly accessible domains, with Cluster 1 showing some extension into the gene body compared to Cluster 2. Cluster 3 shows a narrow region of chromatin accessibility at the TSS, whereas Cluster 4 has a similar enrichment pattern with a moderately lower level of enrichment. Cluster 5 enrichment is lower and broader than Clusters 1–4, and Cluster 6 shows general depletion of ATAC-seq signal.

Given the differences in ATAC-seq enrichment levels and patterns in *Neurospora* promoters, we wanted to know if there was a correlation between promoter structure and transcript level. We plotted the log2-normalized transcripts per million (TPM) of genes within each k-means cluster (Fig. 1D). In general, the average level of expression was higher in clusters with more ATAC-seq accessibility, whereas genes in Cluster 6 had lower transcript levels on average. Although we did observe differences in the average transcript levels between clusters, all clusters had genes with either very high or very low expression indicating that chromatin accessibility in *Neurospora* does not necessarily result in higher mRNA transcription (Fig. 1D). Instead, it is likely that accessible chromatin regions correspond to binding sites of both transcriptional activators and repressors.

We next asked if distinct promoter structures were enriched for any functional categories of genes. In particular, we were interested in genes with large, hyperaccessible chromatin regulatory regions. We used ACRs identified from unsorted nuclei, as described above (*n* = 7132), and we ranked them by size. We annotated ACRs greater than 2 kb (*n* = 84) to their closest genes (Table S2; Fig. 1E) and performed Gene Ontology (GO) analysis. This revealed that clusters of genes with large, hyperaccessible chromatin regions are significantly enriched for GO categories “sequence-specific DNA binding”, “transcription factor activity”, and “regulation of transcription” (Fig. 1F; Table S5). Closer inspection of these genes also reveals that they are involved in cellular processes including light response, conidial separation, and other master regulatory functions, including the carbon catabolite repressor *cre-1,* and a member of the light-response pioneer factor complex WCC, *white collar 1* (*wc-1*).

Because gene annotation software determines annotated genes based on distance from the feature of interest, and these regions could annotate to more than one gene, we were concerned that the annotation software was giving us inaccurate results. We therefore completed the same analysis using Cluster 1 and Cluster 2 from Fig. 1C (Table S4). Our analyses revealed that Cluster 2 is enriched for genes which are annotated to have transcriptional roles (i.e. RNA Polymerase 2 (Pol2) activity, TF binding, and sequence-specific DNA binding), while Cluster 1 is more enriched for signal transduction activity (Fig. S2; Table S5). This supports our finding that genes with large, hyperaccessible chromatin regions are enriched for genes with regulatory functions.

### ACRs are associated with high nucleosome turnover and transcription factor binding

**Fig. 2:**
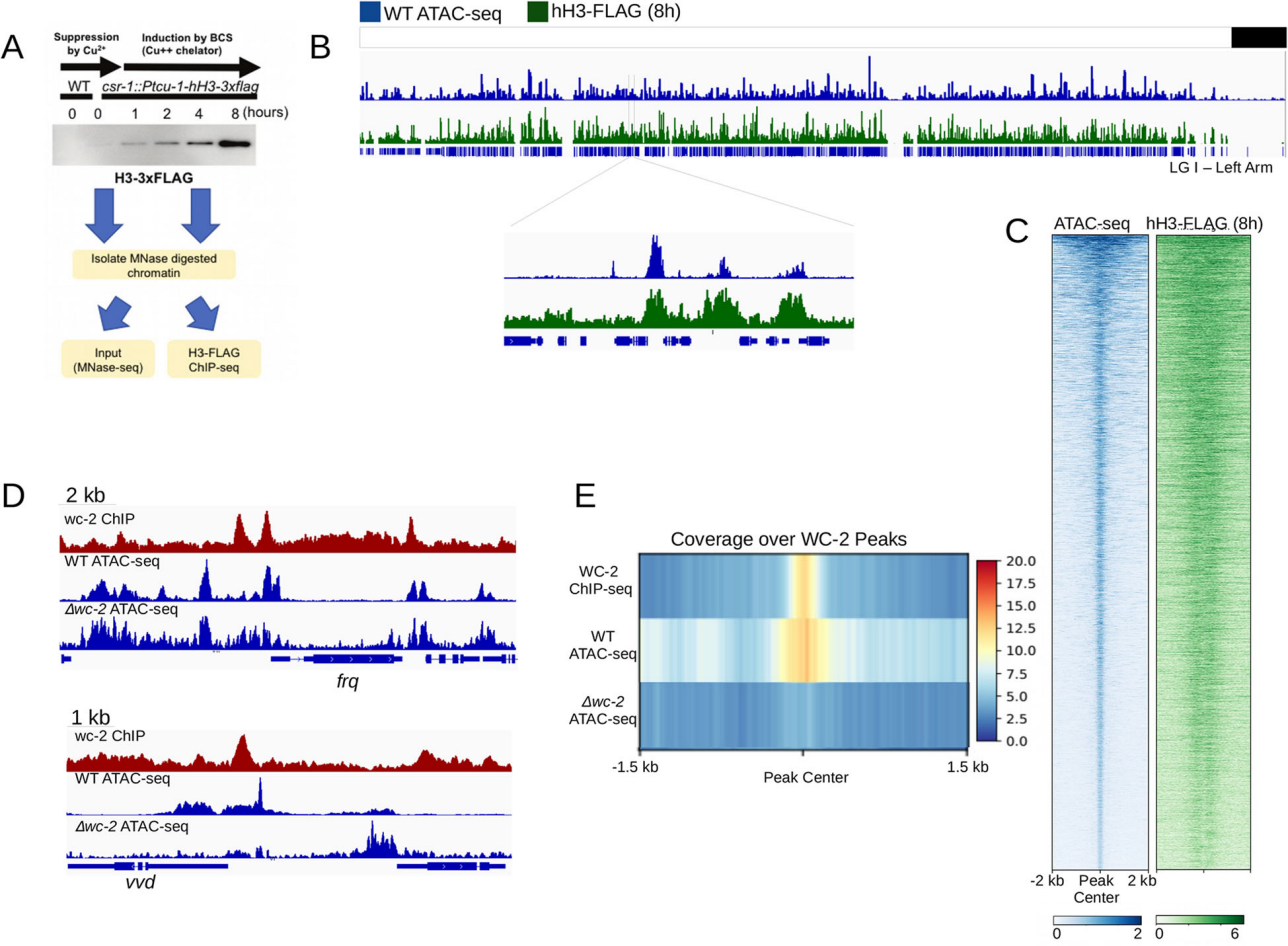
Chromatin accessibility is associated with nucleosome turnover and transcription factor binding. (A) The Western blot shows induction of an ectopic H3-3xFLAG construct under the control of a copper-regulated promoter to measure integration of new H3 (i.e. hH3 turnover). Protein levels are shown at 0, 1, 2, 3, 4, and 8 h after induction using the copper chelator BCS. (B) The genome browser image for the left arm of Linkage Group 1 (LGI-Left Arm) illustrates the overlapping patterns of accessible chromatin (blue) and newly incorporated H3-3xFLAG (green). (C) The heatmap shows global overlap of chromatin accessibility and histone turnover measured by H3-3xflag enrichment following induction. The heatmaps are centered on ACRs and ordered by peak size. (D) Representative genome browser images of the *frq* locus and *vvd* locus illustrate ATAC-seq enrichment patterns in WT and the Δ*wc-2* mutant. (E) The heatmap shows enrichment data across all WC2-binding sites for the indicated experiments. Previously published WC-2 ChIP-seq enrichment is shown for the wild-type strain. ATAC-seq enrichment is shown for wild type and the Δ*wc-2* mutant

Pioneer factors are DNA-binding proteins that can displace nucleosomes to create accessible chromatin regions. To determine if ACRs are associated with higher rates of nucleosome turnover, we examined rates of nucleosome exchange across the *Neurospora* genome using an inducible hH3-3xFLAG protein. We first introduced an H3-3xFLAG fusion protein (*hH3:3xflag*) under the control of a copper-inducible promoter, and we confirmed that the H3-3xFLAG protein is induced under copper limiting conditions and incorporated into chromatin (Fig. 2A). We then performed ChIP-seq following MNase digestion (MNase-ChIP) to identify regions with high H3-3xFLAG enrichment, reasoning that incorporation of newly synthesized H3-3xFLAG would preferentially occur in regions with high rates of nucleosome turnover (i.e. due to competition between TFs and nucleosomes). Conceptually similar approaches have been applied in other fungal systems and recently in *N. crassa* [54–56]. We plotted H3-3xFLAG enrichment after 8 h of induction and found high levels of H3 turnover correspond to ACRs (Figs. 2B, S3). We then plotted nucleosome turnover (i.e. H3-3xFLAG enrichment) across all ACRs, which revealed high nucleosome turnover at these regions (Fig. 2C). We confirmed significant overlap of ATAC-seq peaks and H3-3xFLAG peaks using a permutation test (*p* < 0.001).

We next asked if ATAC-seq could be used to identify pioneer transcription factors in *Neurospora* by asking if disruption of the White Collar Complex (WCC) would lead to altered patterns of accessible chromatin measured by ATAC-seq. Transcription factors have been demonstrated to provide a mechanism for creating chromatin accessibility at gene promoters, and Sancar and colleagues previously implicated WCC in maintenance of accessible chromatin using MNase assays in wild type and strains lacking a functional WCC. Thus, WCC is proposed to function as a pioneer transcription factor [33]⁠. We asked if ATAC-seq data might provide comparable or even more robust results than MNase assays by performing ATAC-seq assays in wild type and the *wc-2* mutant. We plotted ATAC-seq reads across all WCC binding sites [57]⁠⁠ for wild type and the *wc-2* mutant. We observed a striking reduction of chromatin accessibility in the *Δwc-2* mutant at these sites. These data confirm prior work indicating that the WCC functions as a pioneer transcription factor (Fig. 2D,E). To demonstrate that reduced chromatin accessibility at WC-2 binding sites in *Δwc-2* is not an experimental artefact, we compared heatmaps of ATAC-seq data from wild type and Δ*wc-2* and found no global reduction in ATAC-seq read enrichment genome-wide (Fig. S4; Table S6). Taken together, these data suggest that ATAC-seq is a robust method for examining the relationship between transcription factors and chromatin accessibility in filamentous fungi.

### Accessible chromatin is associated with non-coding transcriptional activity in *N. crassa*

**Fig. 3:**
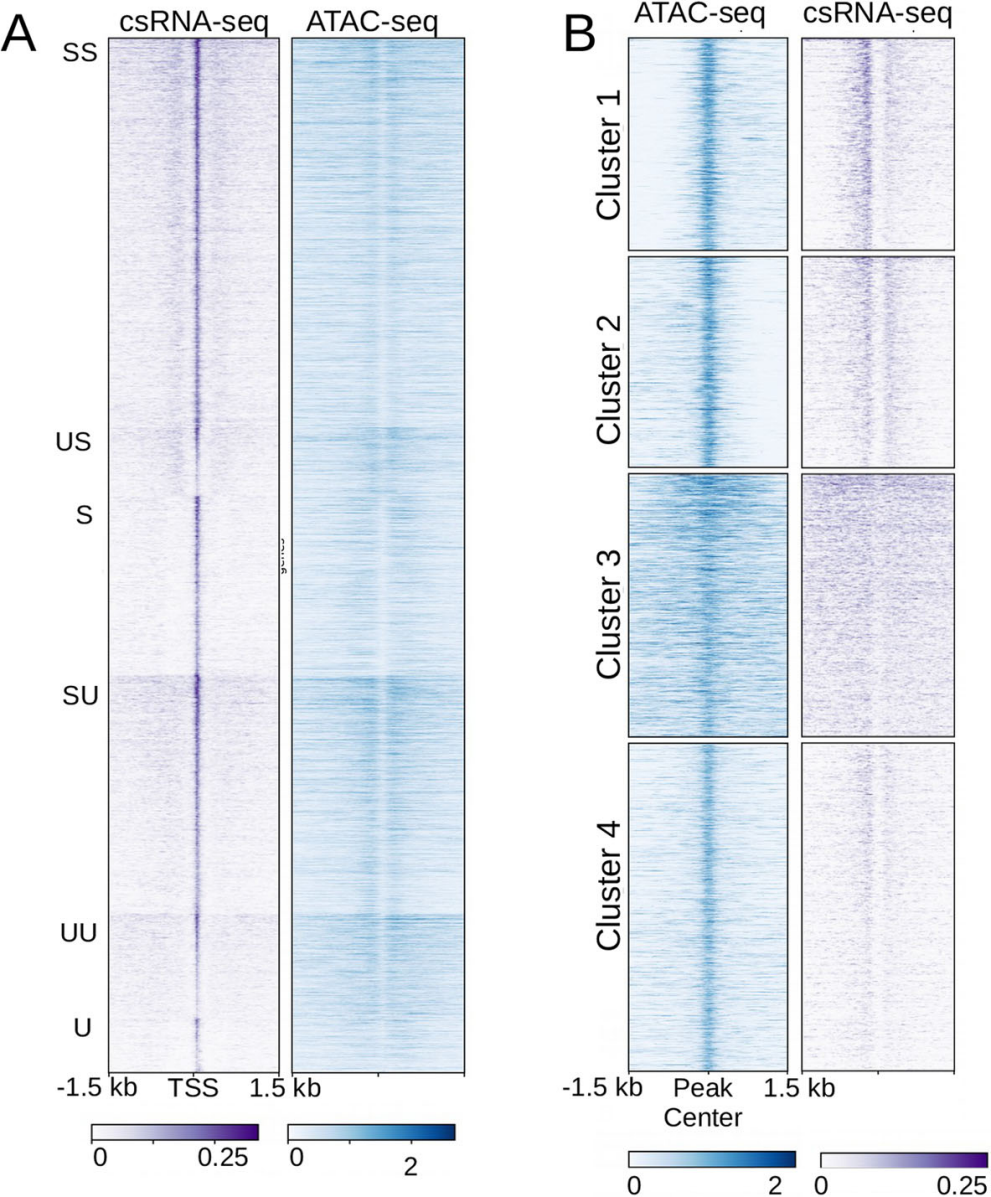
Chromatin accessibility is associated with non-coding transcriptional activity. (A) A heatmap of csRNA-seq reads centered on the TSS of csRNA transcripts ranked by enrichment of each subclass of csRNAs (SS: bi-directional stable; US: unstable pos-strand, stable neg-strand; S: stable unidirectional; SU: stable pos-strand, unstable neg-strand; UU: bidirectionally unstable; U: unidirectional unstable). (B) Heatmap centered on ACRs shows enrichment of csRNAs in relation to accessible chromatin regions

We next wanted to identify additional chromatin features that correlate with accessible chromatin. Recent work identified small abortive transcripts (capped small RNAs; csRNAs) that map to *Neurospora* intergenic regions, raising the possibility that small RNAs or active transcription is a feature of accessible chromatin in filamentous fungi as reported for other eukaryotes [36, 37]⁠. These small RNAs are short (< 60 bp); can be stable or unstable, as determined by read depth when compared to total RNA; and can be uni-directional or bi-directional. To determine if these abortive transcripts are features of accessible regions in *Neurospora,* we compared the previously published csRNA transcript data to our ATAC-seq data. We first asked if regions that generate csRNA transcripts are accessible. We created a heatmap centered on the TSS of csRNAs sorted by stability and directionality as defined above. When we compare these transcripts to chromatin accessibility data, we find that the TSSs of csRNAs are accessible (Fig. 3A). Similarly, a plot of csRNA transcripts across all ACRs reveals that csRNA transcripts occur adjacent to many of them, however not all ACRs contain high levels of csRNAs (Fig. 3B).

To quantify the extent of overlap between chromatin accessibility and csRNA transcripts, we used bedtools intersect to identify ACRs that contain csRNA transcripts (Table S7) and found that 73% of ACRs (*n* = 5213) overlap with at least one annotated csRNA transcript. Conversely, only 60% of csRNA transcripts (*n* = 9709) are found within accessible chromatin regions. Many of these csRNA transcripts appeared to flank accessible chromatin regions. This is a feature that is common among higher eukaryotic promoters and enhancers [58, 59]. We therefore examined enrichment of csRNAs within 100 bp of accessible chromatin in *Neurospora* using bedtools closest. We found that 83% of ACRs either contained or occurred within 100 bp of an annotated csRNA transcript (*n* = 5939). Conversely, approximately 9% of csRNAs were identified within these 100-bp regions (*n* = 1570). In total, 69% of csRNA transcripts either overlapped or were within 100 bp of an ACR (*n* = 11,393). Together, these data indicate that non-coding transcriptional activity is a widespread feature of accessible regions in *Neurospora.*

### H3 acetylation is associated with ACRs and H3K36me3 marks inaccessible chromatin

**Fig. 4:**
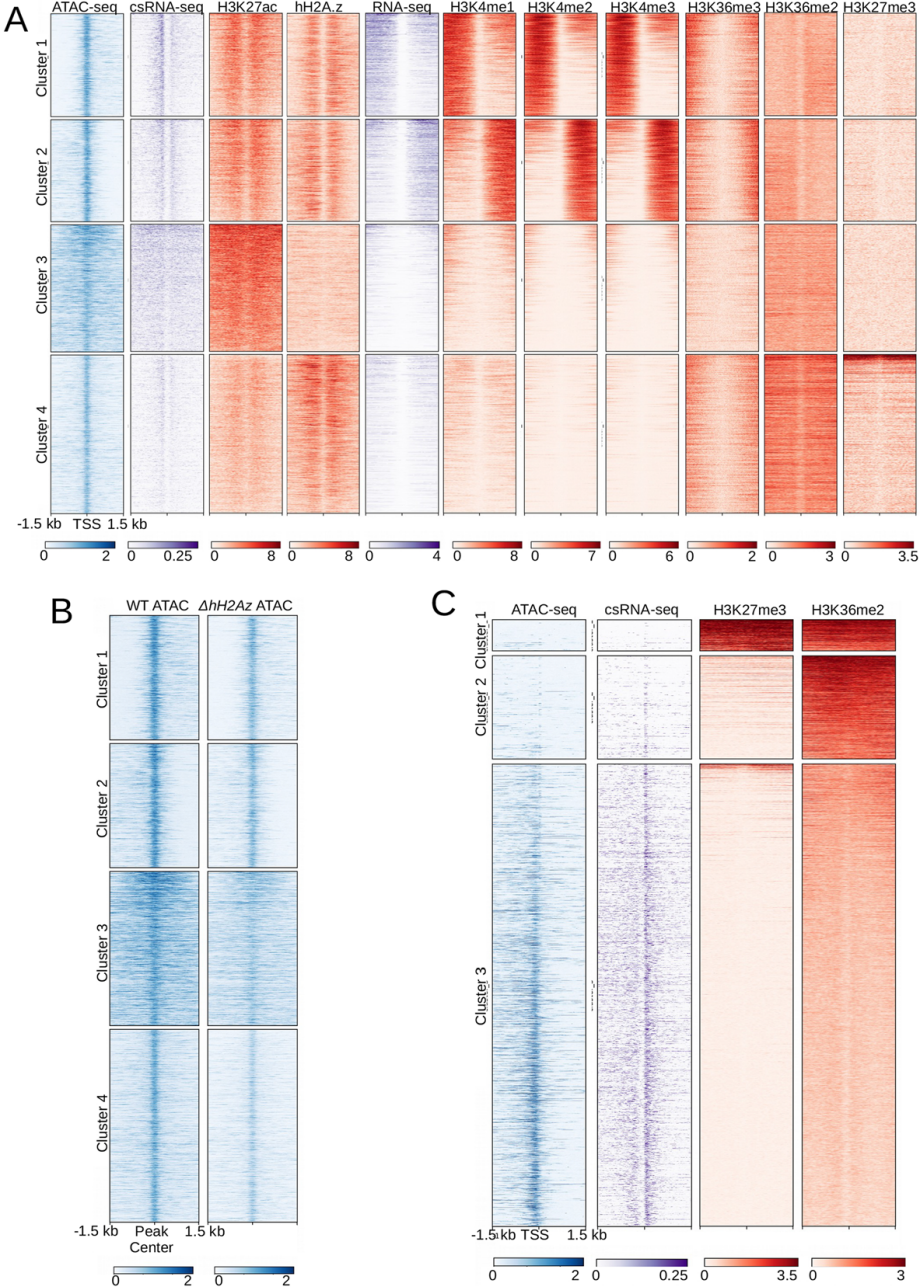
Examination of chromatin features associated with ATAC-seq accessible regions (A) Heatmaps display enrichment for the indicated chromatin feature centered on all ACRs and clustered using k-means (k = 4) to group ACRs with similar chromatin environments. (B) Heatmaps show accessible regions in the wild type and *ΔH2Az* mutant strain Heatmaps are centered on wild type ACRs clustered as in panel A. (C) Heatmap centered on TSSs of genes with unambiguous promoters showing enrichment of chromatin accessibility, csRNAs, H3K27me2/3, and H3K36me2 obtained in a *set-2* mutant (H3K36me2 data are previously published data by Bicocca et al. TSSs were clustered using k-means (k = 3) revealing promoters with K27me2/3 and ASH1-catalyzed H3K36 methylation, promoters with ASH1-catalyzed H3K36methylation alone, and open promoters without either repressive modification

To identify additional chromatin features that are associated with accessible regions, we plotted enrichment of ChIP-seq data for various histone modifications surrounding accessible regions (Fig. 4A). We performed k-means clustering to construct heatmaps using enrichment data for a suite of chromatin features, cs-RNA reads, and mRNA-seq reads to identify chromatin features that are associated with accessible and inaccessible chromatin regions. A heatmap of read density centered on ACRs reveals that virtually all regions of chromatin accessibility are adjacent to nucleosomes containing H3K27ac. The direction of productive transcription is evident by visualizing enrichment of mRNA-seq reads near the ACRs. The heatmap in Fig. 4A further supports the conclusion that accessible chromatin regions are not necessarily associated with active transcription, as shown by the low level of mRNA enrichment in Clusters 3 and 4. H3K4me was correlated with productive transcripts but was not associated with all ACRs.

H2A.Z is reported to promote chromatin accessibility in in embryonic stem cells [60]⁠⁠. We compared previously published H2A.Z ChIP-seq data [22] to our ATAC-seq data and found that H2A.Z was enriched flanking most, but not all ACRs (e.g. see Cluster 3). We then carried out ATAC-seq experiments in wild type and *ΔhH2Az,* which revealed that loss of H2A.Z did not alter global patterns of chromatin accessibility in *Neurospora* (Fig. 4B)*.* Thus, H2A.Z is not a major determinant of accessible chromatin in *Neurospora.*

Recent work has identified a novel role of ASH1-mediated H3K36me2 in gene repression in *Neurospora* [50]⁠⁠. Namely, Bicocca et al. identified regions of the *Neurospora* genome that were both transcriptionally repressed and marked by ASH1-catalyzed H3K36me2 or both H3K27me2/3 and ASH1-catalyzed H3K36me2⁠. We compared previously published enrichment data for ASH1-dependent H3K36me2 with our chromatin accessibility data and found a small number of ACRs that are marked by these repressive histone modifications (Cluster 4 of Fig. 4A). Small regions of chromatin accessibility have been identified in facultative heterochromatin in other organisms. In particular, Polycomb Response Elements (PREs) that function to recruit Polycomb complexes are nucleosome free and accessible, while surrounding regions are inaccessible [61, 62]. We asked if these ACRs were enriched for any specific DNA sequences, which would be consistent with a PRE-like function. We used Hypergeometric Optimization of Motif EnRichment (HOMER) to search for known and unknown DNA motifs enriched under ACRs within PRC2-target domains. Motif analysis did not identify unknown enriched sequences with high confidence (Fig. S5). Interestingly, the top two known motifs identified by HOMER contain a complete or partial identity to the *Neurospora* telomere repeat sequence, TTAGGG, which is sufficient to recruit PRC2 to establish a domain of H3K27me2/3 [63].

Next, we compared the pattern of chromatin accessibility, H3K27me2/3 and ASH1-catalyzed H3K36 di-methylation across the TSSs of genes with unambiguous 5′ regions, as in Fig. 1C. A heatmap centered on the TSS of distinct promoters shows that promoters marked by H3K27me2/3 and H3K36me2 or H3K36me2 alone are highly inaccessible (Fig. 4C). Together, these data demonstrate that H3K36 methylation within euchromatin domains is correlated with inaccessible chromatin in promoters.

## Discussion

We carried out a global analysis of chromatin accessibility in a filamentous fungus using ATAC-seq. Filamentous fungi are important in ecological, industrial, clinical, and evolutionary contexts, and *Neurospora crassa* is an important model for chromatin and epigenetic studies due to shared pathways with higher eukaryotes [9, 16]. Our analyses provide experimental evidence that facultative and constitutive heterochromatin regions marked by H3K27me2/3 and H3K9me3 are largely inaccessible in *Neurospora.* This result is interesting given that fungi lack Polycomb Repressive Complex 1 (PRC1), which is proposed to play a critical role in compacting chromatin within H3K27me3-enriched regions in higher eukaryotes [64, 65]. We also found that promoter chromatin accessibility is not tightly correlated with transcript levels. This suggests that accessible chromatin is occupied by both activators and repressors and therefore should not be used as a measure of active gene expression in filamentous fungi. Conversely, there is a strong correlation between accessible chromatin and H3 turnover. These data support a model in which accessible chromatin results from a competition between promoters and DNA-binding transcription factors. Indeed, we confirm using ATAC-seq that WCC is required to maintain accessible chromatin at WCC binding sites. It is likely that WCC is needed to drive formation of accessible chromatin and allow transcription factors to bind nearby sequences.

Our findings in combination with those of Duttke et al. demonstrate that abortive transcription is a key feature of accessible chromatin in *Neurospora.* The presence of non-coding transcription from distal accessible regions in *Neurospora* is reminiscent of enhancers in higher eukaryotes. Similar results were obtained in the cnidarian *Nematostella* [37, 66] supporting that model that non-coding transcription is an evolutionarily conserved mechanisms of eukaryotic gene regulation. In higher eukaryotes, short unstable RNAs typically flank enhancers and promoters [58, 59]. It is unclear if the small RNAs produced from accessible regions provide a specific function: (1) They could simply be results of promiscuous Pol II activity and themselves be non-functional; (2) These could be regulatory RNAs; or (3) The act of transcription itself may be important in maintaining accessible chromatin in filamentous fungi. Recently, a new model of promoter/enhancer characterization has been proposed in which genomic regulatory elements have enhancer and promoter potential [67]. This model defines promoters and enhancers as regulatory elements at any accessible chromatin region bound by transcription factors, with the ability to recruit Pol II and initiate transcription at either edge, which can positively influence transcription initiation at other regulatory elements. Therefore, it is likely that these RNAs themselves may not be regulatory, but Pol II recruitment to regulatory elements leads to some level of transcription, and the occupancy of Pol II at these sites helps to maintain their accessibility.

We did find a subset of annotated csRNA loci that were not associated with ACRs; however, these may reflect differences in growth conditions used for our ATAC-seq experiments and the csRNA experiments carried out by Duttke et al. Experimental variations including growth conditions, single versus mixed tissue type, and light exposure can produce quite varied results in *Neurospora* expression profiles. It will be important, therefore, to perform more in-depth analysis at these putative *cis*-regulatory regions to determine the role and abundance of these types of RNAs.⁠ Importantly, the growth conditions of the csRNA-seq experiments performed by Duttke et al. vary from our ATAC-seq protocol: csRNA-seq cultures were grown for 2 days at constant light, while cultures used for ATAC-seq were grown overnight at constant light. Therefore, matched experiments will allow for more fine-tuned associations between csRNA production and chromatin accessibility.

The presence of H3K27me2/3 in a small number of accessible chromatin regions also presents an interesting finding, suggesting that PRE equivalents may exist in filamentous fungi. Recent work has identified telomere repeat sequences and the presence of H3K36me2 as prerequisites for H3K27me via PRC2 [63], and a recent study identified PRC2 accessory subunit (PAS) as a PRC2-interacting protein that recruits the complex to a subset of target domains [68]. More work is needed to determine if these ACRs within PRC2-target domains are indeed playing a role in recruitment of PRC2 and establishment of H3K27me.

Our results support the findings reported in Bicocca et al. [50], and suggest that H3K36me may contribute to gene repression by maintaining an inaccessible chromatin structure within silent promoters. Indeed, H3K36 methylation may be a major determinant of inaccessible chromatin within active regions of the genome. Anti-sense transcription at the *Neursopora frequency* (*frq*) locus has been demonstrated to regulate circadian clock function via H3K36me3 [69, 70]⁠. The anti-sense transcript of *frq* (*qrf*) is proposed to stall RNA Polymerase II at the *frq* locus, resulting in premature termination of transcription and establishment of H3K36me3 that precludes binding by the WCC [70]. Our global data are consistent with a repressive role for H3K36me3 when found in regulatory regions. The recent work investigating ASH1-dependent H3K36me in *Neurospora* could provide a model for gene repression in other fungi, especially those that lack PRC2 and H3K27 methylation [71, 72]⁠. *F. fujikuroi* strains lacking ASH1 mediated H3K36me2 exhibited increased expression of secondary metabolism biosynthetic clusters [49]⁠. Despite a lack of ASH1, H3K36me3 also performs a repressive role in *S. cerevisiae* and *S. pombe*, where it is responsible for preventing cryptic transcription and heterochromatic silencing via recruitment of nucleosome remodelers and histone deacetylases [43, 73, 74]. It is possible, therefore, that H3K36me plays a conserved role in gene repression in fungi, namely by contributing to an inaccessible local chromatin architecture within both gene bodies and promoters, as suggested by the work here.

## Conclusions

Profiling accessible chromatin by ATAC-seq uncovered key features of accessible and inaccessible chromatin in the *N. crassa* genome. Accessible chromatin is found in promoters and 5′ regulatory regions of genes. Distinct patterns of accessibility exist, with large, highly accessible intergenic regions frequently associated with master regulatory genes. Accessible regions depend on transcription factor binding and are associated with high rates of nucleosome turnover. H3K27 acetylation is strongly correlated with accessible chromatin, whereas ASH-1-dependent H3K36 methylation, H3K27 methylation, and H3K9 methylation are correlated with inaccessible chromatin.

## Methods

### Culture growth conditions and strain construction

All liquid cultures were grown in Vogel’s minimal medium with 1.5% sucrose with shaking at 32 °C for 18 h. Strains were constructed using standard *Neurospora* protocols [75, 76]⁠⁠. All strains used in this study are listed in Table S8 and primers used for strain construction are listed in Table S9. To construct strain S564 containing a *hH3-3xflag* under control of the copper-regulated *tcu-1* promoter [77], the *hH3* coding sequence was amplified using primers, hH3 inF FP and hH3 inF RP, and then cloned using the In-Fusion HD Cloning HD kit (Clontech) into *Pac*I site of a linearized version of pCCG-3xFLAG plasmid [78]. An *hH3-3xflag* fragment was amplified using primers hH3 CDS FP and hH3 CDS + FLAG RP from the constructed vector (pCCG-C-hH3-3xFLAG). A fragment of the *tcu-1* promoter was amplified using primers *Ptcu-1* FP and Ptcu-1 RP + h3 CDS. To assemble *Ptcu-1-hH3-3xflag*, equal molar amounts of each DNA fragment was mixed and used as a template for PCR with primers, *Ptcu-1* FP and 3xFLAG RP. The *Ptcu-1-hH3-3xflag* was cloned into the pMD19 TA cloning vector.

The *csr-1* locus containing csr-1 5′ flank-CDS-csr-1 3′ flank was amplified by using primers, csr-1 5′ FLANK FP3 and csr-1 3′ FLANK RP3, and cloned into pMD19 TA cloning vector to create plasmid pMD19-CSR-1. To generate the CDS-less version of a linearized version of this vector, inverse PCR was performed using primers csr-1 5 flank RP + Ptcu-1 and csr-1 3 flank FP + FLAG. The *Ptcu-1-hH3-3xflag* fragment was cloned into this linearized vector using In-Fusion cloning. The construct was integrated into the *csr-1* locus by transformation and selection on Cyclosporin A as described [79].

### ATAC-seq

ATAC-seq was performed as described with minor modifications [24, 25]. Briefly, overnight mycelial cultures were lysed in lysis buffer (15 mM Tris pH 7.5; 2 mM EDTA; 0.5 mM spermine; 80 mM KCl; 20 mM NaCl; 15 mM (or 0.1% v/v) β-me; 0.3% TrixtonX-100) by placing mycelia in a petri dish and chopping mycelia with a razor blade. Nuclei were isolated by passing the lysate through miracloth followed by centrifugation at 1000 rpm for 5 min in a microcentrifuge. Nuclei were resuspended in 200 uL of lysis buffer and stored on ice for a maximum of 2 h. To perform transposon integration, nuclei were centrifuged at 1000 rpm for 5 min and washed in 50 uL 1X TAPS buffer (10 mM TAPS-NaOH, 5 mM MgCl2, 10% Dimethylformamide, pH 8.0). After centrifugation, Tn5 integration was performed in 30 uL of tagmentation buffer using modified Tn5 transposase pre-loaded with Illumina sequencing adaptors⁠; samples were incubated for 30 min at 37 °C and fragmented DNA was isolated using the Qiagen MinElute kit (Cat#. 28,004). Sequencing libraries were constructed using Phusion polymerase (ThermoFisher Cat # F549N) and amplified with primers provided in [24]⁠⁠. Libraries were cleaned using SeraPure beads (Fisher Cat. # 09–981-123) before submission for sequencing as described in [80, 81]. Libraries were sequenced on an Illumina NextSeq 500 instrument by Georgia Genomics Facility at University of Georgia.

### Data analysis

ATAC-seq reads were trimmed and quality scored with TrimGalore! (https://github.com/FelixKrueger/TrimGalore). Trimmed reads were mapped to the NC12 genome (GCA_000182925.2_NC12) with BowTie2 using the –very-sensitive option and setting the maximum insert size set at 2000 bp [82].

Analysis of transcriptional start sites (TSSs) was performed on genes with unambiguous regulatory regions. Briefly, a list of promoters that could be unambiguously assigned to a single promoter was generated from a list of all TSSs in the Neurospora genome created with the GenomicRanges package in R [83, 84]. This list was compared to the genome annotation file using bedtools closest to identify the distances between TSSs [85]. Only genes with TSSs that were at least 1500 base pairs apart were classified as genes with unambiguous promoters (*n* = 5200).

ACRs were called using Genrich (https://github.com/jsh58/Genrich)⁠ using the ATAC setting (−j). Reads were separated by insert size using R [84]⁠⁠, and A less than 115 bp were called using MACS2 with a q-value cutoff of 0.01 [86]⁠⁠⁠⁠. FunCat2 [87] was used for genome ontology analysis. Heatmaps and metaplots were made using deepTools [88]⁠⁠. All peak comparisons were performed with bedtools [85]⁠. ACRs were annotated using ChIPseeker [89]⁠.⁠⁠ We used the *peakPermTest* function of the ChIPpeakAnno *R* package to test for significant overlap between ACRs and H3-3xFLAG turnover [90].

Paired-end RNA-seq data was acquired from Joint Genomes Institute Community Sequencing Project 54,089 and were mapped with HISAT2 [91]⁠⁠ using standard options. Counts were created with featureCounts [92]⁠⁠ and analyzed in R [84]. All heatmaps were made using deepTools, and reads were normalized in deepTools for counts per million (CPM) [88].

### Chromatin immunoprecipitation (ChIP-seq)

ChIP-seq was performed as described in [52, 80, 81]⁠ with the following antibodies: H3K27me2/3 (Active Motif Cat# 39535, Lot# 1671401)2; H3K4me1 (Abcam Cat# ab9885); H3K4me2 (Active Motif Cat# 29679, Lot# 31713007); H3K4me3 (Abcam Cat# ab1012); H3K36me3 (Abcam Cat# ab9050, Lot# GR300388–1); H3K9me3 (Active Motif Cat# 39161, Lot# 14418003); H3K27ac; FLAG (Sigma Cat# F1804, Lot# SLBS3530V). Libraries were created and analyzed as described in [81]⁠. Libraries were sequenced by Georgia Genomics Facility at University of Georgia on a NextSeq 500 instrument. Motif analysis was performed with HOMER findMotif.pl using standard options [93]. ChIP-seq peaks were called using MACS2, with a q-value cutoff of 0.01 [86]⁠.

MNase-seq for hH3-3xFLAG was performed in cells eight hours after copper induction. Cells were crosslinked in 1% formaldehyde and transferred to NPS buffer (10 mM Tris-HCl, pH 7.5; 50 mM NaCl; 25 mM MgCl; 1 mM CaCl) and sonicated to shear the chromatin. MNase was performed with 20 U/μl Takara MNase (Cat # 2910A) at 37 °C for 60 min. MNase digestion was quenched with 10 mM EDTA and 100 mM NaCl. Anti-flag ChIP was performed as described above. Previously published data for H3K36me2 in the ∆*set-2* mutant, [50], hH2Az-GFP [22], and WC-2, [57] were re-mapped to the current *Neurospora* genome assembly and analyzed as described above.

### csRNA-seq analysis

csRNA-seq was downloaded from the NCBI Short Read archive and mapped to the NC12 genome (GCA_000182925.2_NC12) and analyzed using csRNA tools developed in HOMER as described in [37]⁠⁠.⁠.

## Supplementary Information

**Additional file 1: Figure S1**. Sorting nuclei does not fully remove mitochondrial contamination. (A) Bar chart of mapped reads per chromosome. Sorted and Unsorted nuclei both exhibit reads mapped to mitochondrial contigs (chromosomes beginning with KC or KI). (B) Heatmap of ATAC-seq enrichment surrounding ACRs called from unsorted nuclei. Patterns of enrichment do not change in sorted versus unsorted nuclei. (C) Representative browser shot of the left arm for LG I showing overlap of sorted and unsorted nuclei ATAC-seq experiments.

**Additional file 2: Figure S2**. GO analysis of genes from Cluster 1 and Cluster 2 in Fig. 1C. Gene Ontology (GO) analysis results for genes whose TSSs fall in either Cluster 1 or Cluster 2 in Fig. 1C

**Additional file 3: Figure S3**. Uncropped blot from Fig. 2B. Lanes 1–6 correspond to those described in the Fig. 2A legend.

**Additional file 4: Figure S4**. Comparison of ATAC-seq experiments in wild type and *Δwc-2*. Heatmap showing ATAC-seq enrichment of all ACRs in wild type and *Δwc-2*. Heatmap is centered on wild type ACRs.

**Additional file 5: Figure S5**. Motif analysis results. Both known and de novo motif analyses of ACRs within H3K27me2/3 regions as determined by HOMER.

**Additional file 6.**

**Additional file 7.**

**Additional file 8.**

**Additional file 9.**

**Additional file 10.**

**Additional file 11.**

**Additional file 12.**

**Additional file 13.**

**Additional file 14.**

## Data Availability

The new datasets supporting the conclusions of this article are available in the NCBI Gene Expression Omnibus (GEO) repository, accession number GSE154497. csRNA-seq was downloaded from the NCBI Short Reach Archive, accession #s: SRR9916743, SRR9916744, SRR91745, SRR9916746, SRR916747, SRR916748, SRR916749, SRR916750. Previously published ChIP-seq data for H3K36me2, hH2A.Z-GFP, and WC-2 were downloaded from the NCBI Short Reach Archive, accession #s: SRS3667122, SRS3667122, SRR1578072. RNA-seq data from wild type were obtained from the Joint Genome Institute Genome Portal (https://genome.jgi.doe.gov/portal/DetofExpression/DetofExpression.info.html).

## Abbreviations

H3Kac: Histone H3 acetylation
H3K4me1/2/3: Histone H3 lysine 4 methylation
H3K36me3: Histone H3 lysine 36 trimethylation
H3K9me3: H3 lysine 9 methylation
H3K27me2/3: Histone H3 lysine 27 methylation
ATAC-seq: Assay for Transposase-Accessible Chromatin sequencing
WCC: White collar complex
*wc-2*: White collar 2
*wc-1*: *White collar 1*
SWI/SNF: Switch/Sucrose non-fermentable
PRC2: Polycomb Repressive Complex 2
TSS: Transcription start site
ACRs: Accessible chromatin regions
TPM: Transcripts per million
GO: Gene Ontology
Pol2: RNA Polymerase 2
MNase-ChIP: ChIP-seq following MNase digestion
csRNAs: Capped small RNAs
PREs: Polycomb Response Elements
HOMER: Hypergeometric Optimization of Motif EnRichment
PRC1: Polycomb Repressive Complex 1
PAS: PRC2 accessory subunit
*frq*: *Frequency*
*qrf*: Anti-sense transcript of *frq*
