# Supplementary figures and images for: Chromatin accessibility profiling in Neurospora crassa reveals molecular features associated with accessible and inaccessible chromatin
Source: BMC Genomics. 2021 Jun 19;22:459. doi: 10.1186/s12864-021-07774-0 (PMC8214302; doi:10.1186/s12864-021-07774-0)

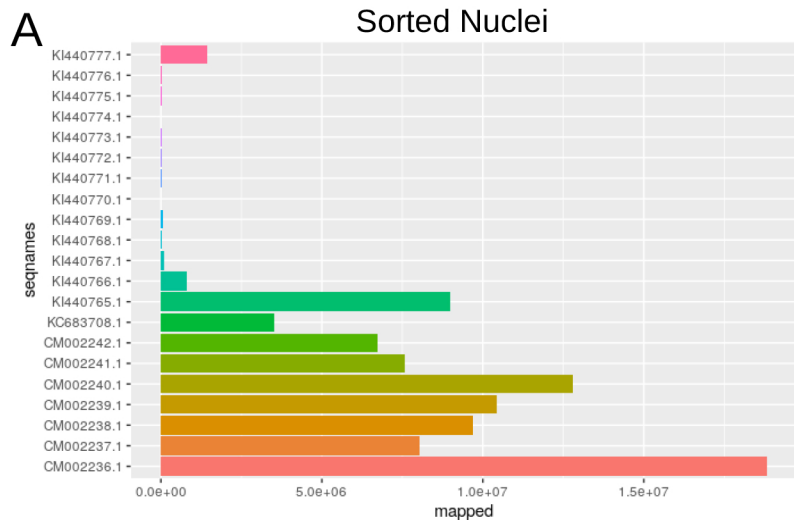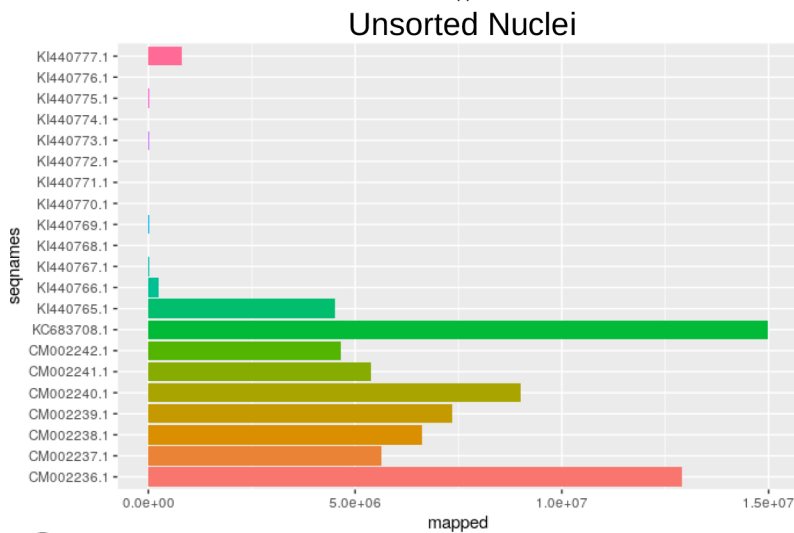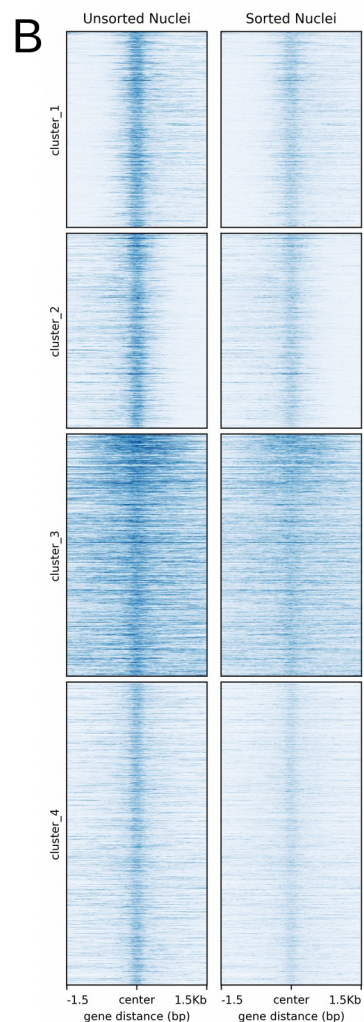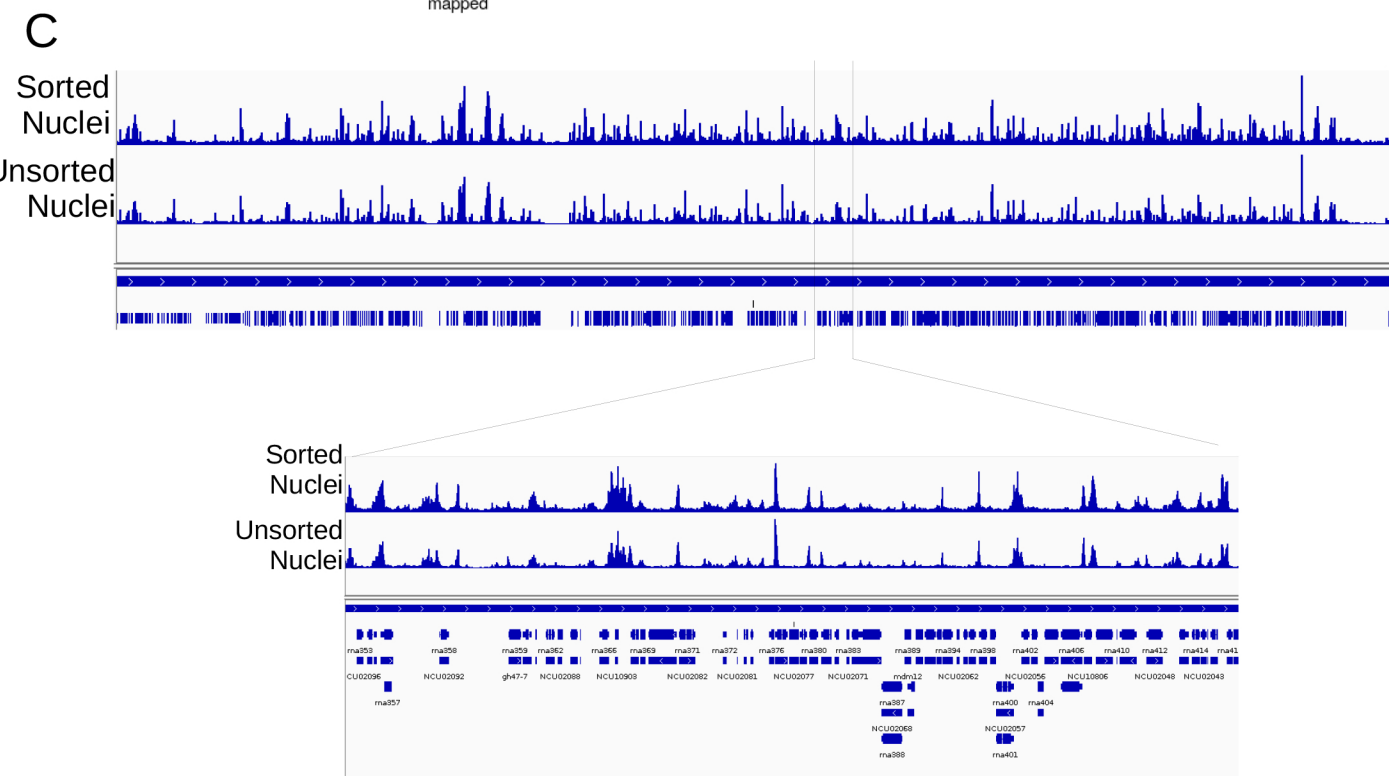

Supplement: Supplementary file 1 — Additional file 1: Figure S1. Sorting nuclei does not fully remove mitochondrial contamination. (A) Bar chart of mapped reads per chromosome. Sorted and Unsorted nuclei both exhibit reads mapped to mitochondrial contigs (chromosomes beginning with KC or KI). (B) Heatmap of ATAC-seq enrichment surrounding ACRs called from unsorted nuclei. Patterns of enrichment do not change in sorted versus unsorted nuclei. (C) Representative browser shot of the left arm for LG I showing overlap of sorted and unsorted nuclei ATAC-seq experiments. [file 12864_2021_7774_MOESM1_ESM.pdf]

# Cluster 1 GO Results

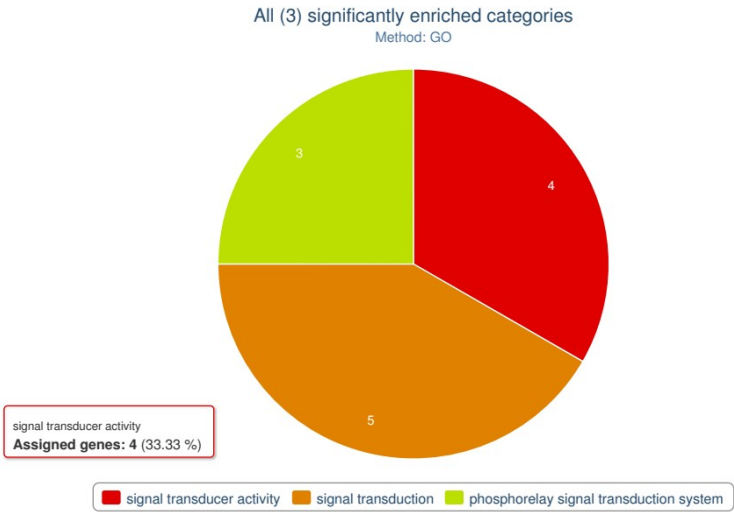

# Cluster 2 GO Results

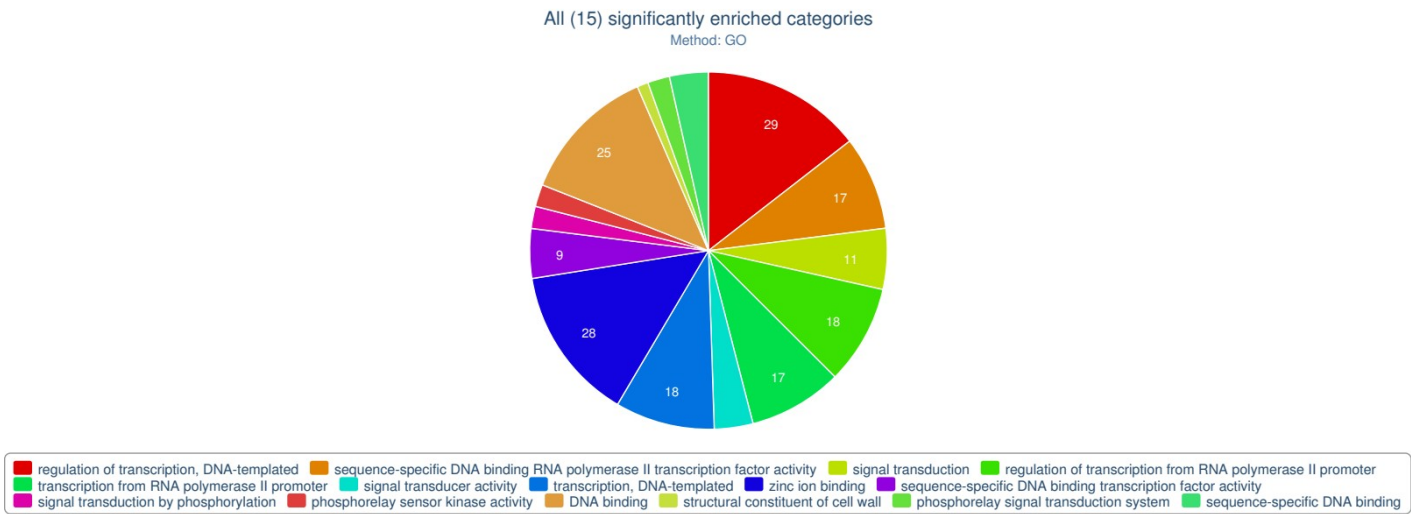

Supplement: Supplementary file 2 — Additional file 2: Figure S2. GO analysis of genes from Cluster 1 and Cluster 2 in Fig. 1C. Gene Ontology (GO) analysis results for genes whose TSSs fall in either Cluster 1 or Cluster 2 in Fig. 1C [file 12864_2021_7774_MOESM2_ESM.pdf]

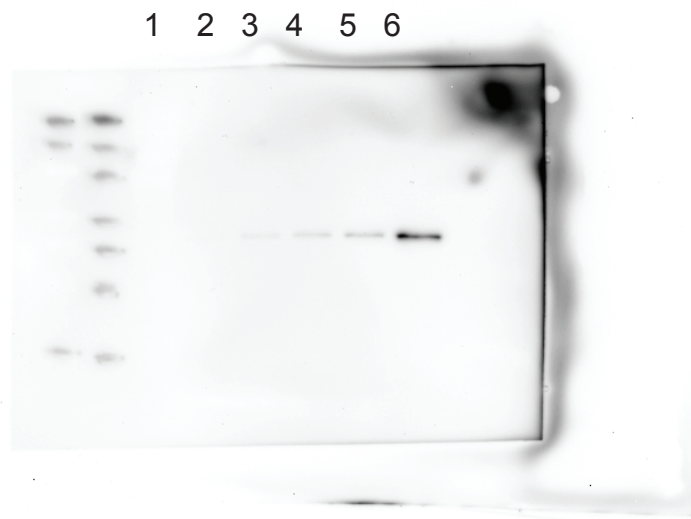

Figure S3. Uncropped blot from Figure 2B. Lanes 1 - 6 correspond those described in the Fig 2A legend.

Supplement: Supplementary file 3 — Additional file 3: Figure S3. Uncropped blot from Fig. 2B. Lanes 1–6 correspond to those described in the Fig. 2A legend. [file 12864_2021_7774_MOESM3_ESM.pdf]

WT ATAC-seq    $\Delta wc-2$  ATAC-seq

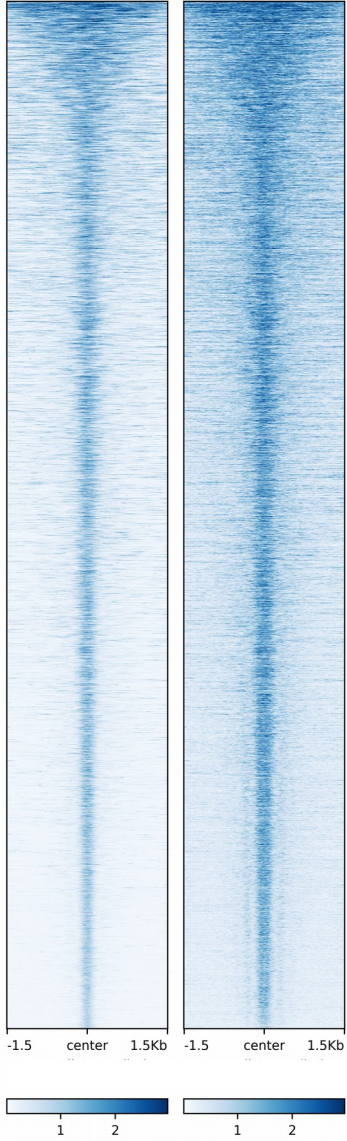

Supplement: Supplementary file 4 — Additional file 4: Figure S4. Comparison of ATAC-seq experiments in wild type and Δwc-2. Heatmap showing ATAC-seq enrichment of all ACRs in wild type and Δwc-2. Heatmap is centered on wild type ACRs. [file 12864_2021_7774_MOESM4_ESM.pdf]
