# Supplementary material for: Chromatin accessibility profiling in Neurospora crassa reveals molecular features associated with accessible and inaccessible chromatin
Source: BMC Genomics. 2021 Jun 19;22:459. doi: 10.1186/s12864-021-07774-0 (PMC8214302; doi:10.1186/s12864-021-07774-0)
Supplement: Supplementary file 5 — Additional file 5: Figure S5. Motif analysis results. Both known and de novo motif analyses of ACRs within H3K27me2/3 regions as determined by HOMER. [file 12864_2021_7774_MOESM5_ESM.pdf]

HOMER Known Motif Results

Rank	Motif	Name	P-value	log P-pvalue	q-value (Benjamini)	# Target Sequences with Motif	% of Targets Sequences with Motif	# Background Sequences with Motif	% of Background Sequences with Motif
1	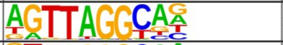	MYB116(MYB)/colamp-MYB116-DAP-Seq(GSE60143)/Homer	1e-4	-9.282e+00	0.0936	20.0	29.41%	5228.8	11.96%
2	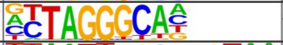	TBP3(MYBrelated)/col-TBP3-DAP-Seq(GSE60143)/Homer	1e-3	-8.480e+00	0.1044	14.0	20.59%	3036.1	6.94%
3	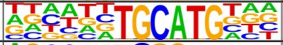	FUS3(ABI3VP1)/col-FUS3-DAP-Seq(GSE60143)/Homer	1e-3	-7.735e+00	0.1467	16.0	23.53%	4087.5	9.35%
4	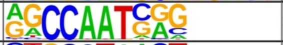	NFY(CCAAT)/Promoter/Homer	1e-3	-7.553e+00	0.1467	17.0	25.00%	4586.3	10.49%
5	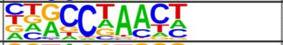	BOS1(MYB)/col-BOS1-DAP-Seq(GSE60143)/Homer	1e-3	-6.914e+00	0.1999	22.0	32.35%	7219.0	16.51%
6	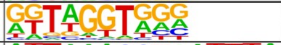	MYB3(MYB)/Arabidopsis-MYB3-ChIP-Seq(GSE80564)/Homer	1e-2	-6.736e+00	0.1999	33.0	48.53%	13228.9	30.26%
7	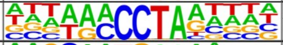	AT1G72740(MYBrelated)/colamp-AT1G72740-DAP-Seq(GSE60143)/Homer	1e-2	-6.579e+00	0.1999	15.0	22.06%	4106.4	9.39%
8	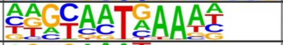	Hoxd12(Homeobox)/ChickenMSG-Hoxd12.Flag-ChIP-Seq(GSE86088)/Homer	1e-2	-6.520e+00	0.1999	17.0	25.00%	5027.2	11.50%
9	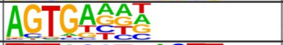	At5g04390(C2H2)/col200-At5g04390-DAP-Seq(GSE60143)/Homer	1e-2	-6.122e+00	0.2453	48.0	70.59%	23112.9	52.86%
10	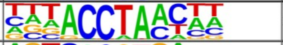	MYB121(MYB)/col-MYB121-DAP-Seq(GSE60143)/Homer	1e-2	-6.108e+00	0.2453	16.0	23.53%	4759.4	10.89%
11	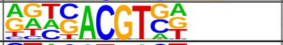	HY5(bZIP)/colamp-HY5-DAP-Seq(GSE60143)/Homer	1e-2	-5.988e+00	0.2453	16.0	23.53%	4815.6	11.01%
12	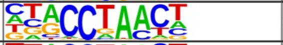	MYB62(MYB)/colamp-MYB62-DAP-Seq(GSE60143)/Homer	1e-2	-5.955e+00	0.2453	27.0	39.71%	10410.0	23.81%
13	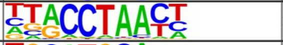	MYB57(MYB)/col-MYB57-DAP-Seq(GSE60143)/Homer	1e-2	-5.384e+00	0.3551	13.0	19.12%	3734.1	8.54%
14	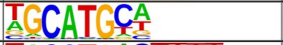	RBFox2(?)/Heart-RBFox2-CLIP-Seq(GSE57926)/Homer	1e-2	-5.180e+00	0.4044	25.0	36.76%	9865.3	22.56%
15	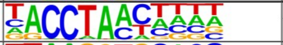	AT3G10580(MYBrelated)/colamp-AT3G10580-DAP-Seq(GSE60143)/Homer	1e-2	-5.149e+00	0.4044	16.0	23.53%	5241.6	11.99%
16	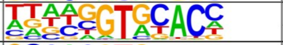	AT3G51470(DBP)/col-AT3G51470-DAP-Seq(GSE60143)/Homer	1e-2	-4.996e+00	0.4254	24.0	35.29%	9455.9	21.63%
17	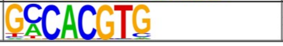	E-box/Arabidopsis-Promoters/Homer	1e-2	-4.941e+00	0.4254	7.0	10.29%	1444.0	3.30%

HOMER *de novo* Motif Results

* - possible false positive

Rank	Motif	P-value	log P-pvalue	% of Targets	% of Background	STD(Bg STD)	Best Match/Details
1 *	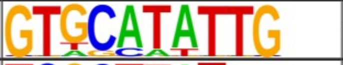	1e-10	-2.323e+01	11.76%	0.33%	58.2bp (72.2bp)	byn/dmmpmm(SeSiMCMC)/fly(0.668)<br>More Information | Similar Motifs Found
2 *	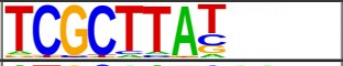	1e-9	-2.291e+01	25.00%	3.44%	49.9bp (76.3bp)	BARHL1/MA0877.2/Jaspar(0.718)<br>More Information | Similar Motifs Found
3 *	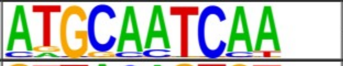	1e-9	-2.110e+01	11.76%	0.43%	61.6bp (68.8bp)	Dux/MA0611.1/Jaspar(0.809)<br>More Information | Similar Motifs Found
4 *	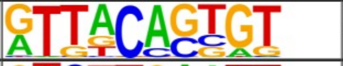	1e-8	-2.065e+01	23.53%	3.45%	64.3bp (77.7bp)	PH0158.1_Rhox11_2/Jaspar(0.681)<br>More Information | Similar Motifs Found
5 *	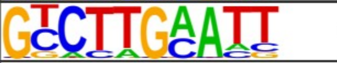	1e-8	-2.001e+01	16.18%	1.35%	61.4bp (71.3bp)	SNRNP70(RRM)/Homo_sapiens-RNCMPT00070-PBM/HughesRNA(0.675)<br>More Information | Similar Motifs Found
6 *	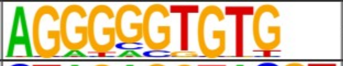	1e-8	-1.889e+01	11.76%	0.57%	57.9bp (73.8bp)	KLF10(Zf)/HEK293-KLF10.GFP-ChIP-Seq(GSE58341)/Homer(0.850)<br>More Information | Similar Motifs Found
7 *	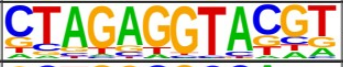	1e-7	-1.637e+01	35.29%	10.79%	50.7bp (88.1bp)	SD0001.1_at_AC_acceptor/Jaspar(0.679)<br>More Information | Similar Motifs Found
8 *	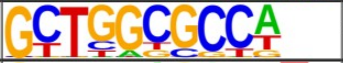	1e-5	-1.351e+01	14.71%	2.06%	48.0bp (68.3bp)	brk/dmmpmm(Papatsenko)/fly(0.778)<br>More Information | Similar Motifs Found
9 *	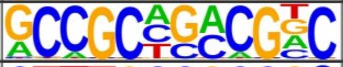	1e-4	-1.044e+01	4.41%	0.09%	47.1bp (57.0bp)	ERF5/MA1225.1/Jaspar(0.738)<br>More Information | Similar Motifs Found
10 *	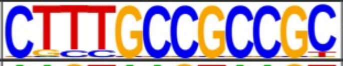	1e-2	-6.467e+00	1.47%	0.00%	0.0bp (22.7bp)	RAP26/MA1221.1/Jaspar(0.777)<br>More Information | Similar Motifs Found
11 *	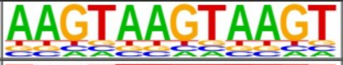	1e-2	-5.774e+00	1.47%	0.01%	6.8bp (10.2bp)	PH0146.1_Pou3f1/Jaspar(0.662)<br>More Information | Similar Motifs Found
12 *	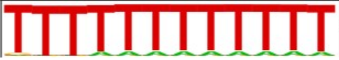	1e0	-1.500e+00	1.47%	0.37%	2.6bp (50.0bp)	SeqBias: polyA-repeat(0.913)<br>More Information | Similar Motifs Found
