# Supplementary material for: Chromatin accessibility profiling in Neurospora crassa reveals molecular features associated with accessible and inaccessible chromatin
Source: BMC Genomics. 2021 Jun 19;22:459. doi: 10.1186/s12864-021-07774-0 (PMC8214302; doi:10.1186/s12864-021-07774-0)
Supplement: Supplementary file 6 — Additional file 6. [file 12864_2021_7774_MOESM6_ESM.docx]

**Table S1 Mapping statistics of ATAC-seq in sorted and unsorted nuclei**

| Sample | Total Reads | Mapped Reads | % Mapped Reads | Chromosomal reads | Mitochondrial Reads | % Mitochondrial Reads |
| --- | --- | --- | --- | --- | --- | --- |
| Sorted | 41,725,868 | 41,0725,868 | 98.4392 | 35,587,105 | 6,138,763 | 14.7121 |
| Unsorted | 34,429,284 | 33,905,374 | 98.4783 | 25,119,456 | 9,609,828 | 27.0404 |
