# Supplementary material for: Chromatin accessibility profiling in Neurospora crassa reveals molecular features associated with accessible and inaccessible chromatin
Source: BMC Genomics. 2021 Jun 19;22:459. doi: 10.1186/s12864-021-07774-0 (PMC8214302; doi:10.1186/s12864-021-07774-0)
Supplement: Supplementary file 13 — Additional file 13. [file 12864_2021_7774_MOESM13_ESM.docx]

**Table S1. Strains used in this study**

| **Strain ID** | **Genotype** |
| --- | --- |
| **FGSC4200** | Wild type |
| **S630** | *hH2A.z::hyg* |
| **FGSC11124** | *wc-2::hyg* |
| **S564** | *csr-1::pTCU-1:hH3:3x-FLAG:hyg* |
