# Supplementary material for: Chromatin accessibility profiling in Neurospora crassa reveals molecular features associated with accessible and inaccessible chromatin
Source: BMC Genomics. 2021 Jun 19;22:459. doi: 10.1186/s12864-021-07774-0 (PMC8214302; doi:10.1186/s12864-021-07774-0)
Supplement: Supplementary file 14 — Additional file 14. [file 12864_2021_7774_MOESM14_ESM.docx]

| Table S9. Primers used in this study. | |
| --- | --- |
| Primer Name | Primer Sequence |
| hH3 inF FP | GATCCCCGGGTTAATATGGCCCGCACTAAGCAGACCGCCC |
| hH3 inF RP | TTTGTAGTCTTTACCGTTGCGCTCACCGCGGAGGCGGCGG |
| 3xFLAG FP | GACTACAAAGACCATGACGGTGAT |
| 3xFLAG RP | CTTGTCATCGTCATCCTTGTAGTC |
| hH3 CDS FP | ATGGCCCGCACTAAGCAGACCGC |
| hH3 CDS+FLAG RP | ATGGTCTTTGTAGTCGTTGCGCTCACCGCGGAGGC |
| Ptcu-1 FP | GATGGGATAGAGAGAATGGC |
| Ptcu-1 RP + h3 CDS | GCGGTCTGCTTAGTGCGGGCCATGGTTGGGGATGTGTGTGC |
| csr-1 5' FLANK FP3 | CAGACATGGCCATCAAGGTCGT |
| csr-1 3' FLANK RP3 | TGTCAAGGTCAATGGGCGCAAG |
| csr-1 5 flank RP + Ptcu-1 | TCTCTCTATCCCATCTTTCACTAAAATGGAAAGGAG |
| csr-1 3 flank FP + FLAG | GATGACGATGACAAGATTCCCTTGTGGACATTGTC |
